# *Lactobacillus paracasei* WIS43 alleviates DSS-induced colitis by modulating gut microbiota and suppressing inflammation

**DOI:** 10.3389/fmicb.2025.1721585

**Published:** 2026-01-29

**Authors:** Yu Fu, Shuxia Chen, Lei Cui, Ying Cao, Yanan Dong, Yunfeng Duan, Chongming Wu

**Affiliations:** 1 School of Chinese Materia Medica, Tianjin University of Traditional Chinese Medicine, Tianjin, China; 2 PKUMed-Wisbiom Joint Laboratory for Human Microbiome Research, Institute of Advanced Clinical Medicine, Peking University, Beijing, China; 3 Department of Pharmacy, Tianjin Jizhou District People’s Hospital, Tianjin, China; 4 Tianjin Key Laboratory of Therapeutic Substance of Traditional Chinese Medicine, Tianjin, China; 5 State Key Laboratory of Chinese Medicine Modernization, Tianjin, China

**Keywords:** gut microbiota, inflammatory cytokines, *Lactobacillus paracasei* WIS43, probiotics, ulcerative colitis

## Abstract

**Introduction:**

Ulcerative colitis (UC) is a chronic inflammatory disorder of the colon with rising incidence and limited therapeutic options. Probiotics are increasingly recognized as potential interventions, but strain-specific differences remain insufficiently defined.

**Methods:**

We conducted a meta-analysis of publicly available microbiome datasets to characterize disease-associated dysbiosis, focusing on the genus *Lactobacillus*. We then evaluated *Lactobacillus paracasei* WIS43, a novel strain isolated from the breast milk of a healthy volunteer, in a dextran sulfate sodium (DSS)-induced murine colitis model, using mesalazine and the commercial strain *Lactobacillus paracasei* LPC-37 as comparators. Disease severity, histopathology, inflammatory cytokines, and gut microbiota composition were systematically assessed.

**Results:**

Meta-analysis confirmed a significant depletion of *Lactobacillus* in UC patients. *In vivo*, WIS43 treatment reduced body weight loss, disease activity index scores, and colon shortening. Histological analysis revealed preserved epithelial integrity and reduced inflammatory infiltration. WIS43 significantly decreased serum and colonic TNF-*α*, IL-6, and IL-1β levels, demonstrating stronger anti-inflammatory activity than LPC-37 and comparable efficacy to mesalazine. 16S rRNA sequencing further showed that WIS43 restored beneficial taxa, including *Lactobacillus johnsonii* and *Lactobacillus taiwanensis*, while reducing potentially pathogenic bacteria.

**Conclusion:**

These findings identify WIS43 as a promising probiotic candidate for the prevention and treatment of UC, supporting its therapeutic potential through coordinated modulation of host immunity and gut microbiota.

## Graphical abstract

**Figure:**
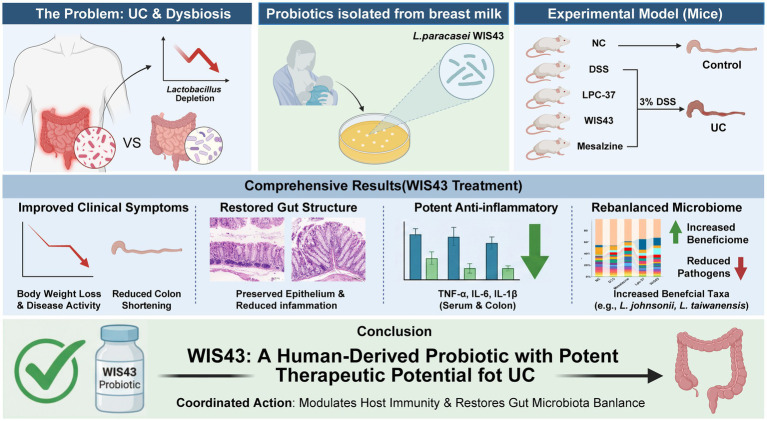

## 1 Introduction

Ulcerative colitis (UC) is a chronic, nonspecific form of inflammatory bowel disease (IBD) that primarily affects the colonic mucosa and submucosa, often involving the rectum and sigmoid colon (Kobayashi et al., 2020). As a chronic inflammatory disorder of unclear etiology, UC has shown a persistently rising global incidence in recent decades. It is projected that by 2035, the prevalence of UC in Asian populations will be four times higher than current levels, thereby imposing a substantial burden on global healthcare systems (Le Berre et al., 2023). The pathogenesis of UC is highly complex and involves the interplay of genetic susceptibility (Du and Ha, 2020), aberrant immune activation (Sham et al., 2018), environmental triggers (Zhang et al., 2015), and gut microbiota dysbiosis (Ekstedt et al., 2024), which together drive chronic mucosal inflammation. Clinically, UC patients often experience recurrent diarrhea, severe abdominal pain, and mucus-purulent-bloody stools (Nakase et al., 2021). These symptoms not only severely compromise quality of life but also markedly increase the long-term risk of colorectal cancer, representing a serious threat to both physical and mental health.

Increasing attention has been directed toward the role of the gut microbiota in the pathogenesis and treatment of UC. Numerous studies have demonstrated that UC patients exhibit profound alterations in gut microbial diversity, composition, and metabolic functions, characterized by a reduction in beneficial bacteria, increased opportunistic pathogens, and decreased production of microbial metabolites such as short-chain fatty acids (SCFAs) (Lavelle and Sokol, 2020). Notably, the transplantation of fecal microbiota from UC patients into germ-free mice induces colitis-like phenotypes, further supporting its pathogenic role (Yang et al., 2022). Conversely, targeted supplementation with probiotics, prebiotics, or microbial metabolites has been shown to alleviate colitis in animal models by restoring microbial balance, enhancing barrier integrity, and modulating host immunity (Cheng et al., 2022; Gu et al., 2023). Traditional Chinese medicine has also demonstrated therapeutic potential via microbiota modulation. For instance, active compounds from *Coptis chinensis* and *Phellodendron amurense* enriched the anti-inflammatory commensal *Blautia producta* and alleviated damp-heat type UC by suppressing TNF-*α*, IL-6, and IL-1β (Zhan et al., 2025). *Houttuynia cordata* was identified as a novel prebiotic candidate that promoted the growth of *Bacteroides thetaiotaomicron* (Zou et al., 2024), while polyphenols from *Stragalus zaissanensis* enriched *Alistipes*, particularly *Alistipes onderdonkii*, thereby enhancing tryptophan metabolism and activating the AhR–IL-22 axis to repair the intestinal barrier (Zhong et al., 2024). Moreover, Qingjie Fuzheng Granule inhibited colitis-associated colorectal cancer progression by inducing macrophage polarization (Huang et al., 2025), and Banxia Xiexin Decoction attenuated inflammation and tumorigenesis in azoxymethane/DSS (dextran sulfate sodium)-induced colorectal cancer via the correction of dysbiosis and suppression of STAT3 activation (Yue et al., 2025). Together, these findings highlight the pivotal role of the gut microbiota in UC and underscore the therapeutic potential of microbiota-targeted interventions.

Probiotics—live microorganisms that, when administered in adequate amounts, confer a health benefit on the host—have attracted considerable interest for their ability to maintain gut microbial homeostasis, enhance intestinal barrier function, and modulate immune responses, thereby offering new therapeutic avenues for ulcerative colitis (Sanders et al., 2019). The most frequently investigated probiotics belong to the genera *Bifidobacterium* and *Lactobacillus*, as well as the species *Streptococcus thermophilus*. For example, *Bifidobacterium pseudolongum* ATCC 25526 alleviated dextran sulfate sodium (DSS)-induced colitis by producing R-equol to promote Foxp3 + T cells (Li M. et al., 2024), while *Bifidobacterium adolescentis* improved mucosal structure and inflammation in chronic colitis through activation of Th2/Treg immunity (Fan et al., 2021). Yogurt fermented with *Lactobacillus bulgaricus* 151 and *S. thermophilus* MK-10 has been shown to enhance IgA production and restore Th1/Th2 balance, thereby ameliorating colitis symptoms (Wasilewska et al., 2019). Similarly, S*. thermophilus* ST28 reduced Th17 cells in DSS-induced colitis (Ogita et al., 2011). Species of the genus *Lactobacillus* have been extensively investigated owing to their well-documented efficacy, genetic stability, safety profile, and ability to survive gastrointestinal transit (Jang et al., 2019). For instance, *Lactobacillus gasseri* JM1 attenuated colitis in mice by regulating tight junction proteins and modulating cytokine expression (Cheng et al., 2022), whereas *Lactobacillus rhamnosus* CY12 restored barrier integrity and suppressed NF-κB signaling (Nascimento et al., 2020).

Among *Lactobacillus* species, *Lactobacillus paracasei* has emerged as a versatile probiotic with broad functional potential, encompassing antioxidant activity, metabolic regulation, immune enhancement, respiratory and oral health benefits, and even neuropsychological improvement. For example, *L. paracasei* LP33 was validated in randomized controlled trials for allergic rhinitis, where it significantly reduced nasal and ocular symptoms and improved quality of life (Güvenç et al., 2016). *L. paracasei* DTA81 lowered cholesterol and fasting blood glucose in diet-induced obese mice (Tarrah et al., 2021), and *L. paracasei* NCU-04 improved constipation-associated depressive-like behavior through serotonin-mediated mechanisms (Li S. et al., 2024). Mechanistically, various *L. paracasei* strains alleviate intestinal inflammation via distinct pathways: BNCC345679 restored beneficial taxa and upregulated tight junction proteins MUC2 and ZO-1 (Ahmad et al., 2024); strain R3 regulated Th17/Treg balance (Huang et al., 2021); and heat-inactivated *L. paracasei* repaired LPS-induced barrier injury through the MLCK/MLC pathway (Xie et al., 2023). However, probiotic efficacy is widely acknowledged to be strain-specific (Gill and Guarner, 2004), with different *L. paracasei* strains exhibiting variable anti-inflammatory capacities, adhesion abilities, and immunomodulatory potential. Thus, functional attributes cannot be generalized across the species, and expanding the strain repertoire remains critical. Although several studies have reported the beneficial effects of *L. paracasei* in alleviating experimental colitis—primarily through immunomodulation or barrier protection—current research still has notable limitations. Most studies lack direct comparisons with standard drugs or other probiotic strains, making it difficult to clarify strain-specific advantages. In addition, many microbiota analyses rely on short-read sequencing with limited taxonomic resolution, which hampers precise identification of key functional taxa. Therefore, more systematic studies are needed to overcome these limitations and provide robust evidence for the therapeutic potential of novel *L. paracasei* strains.

In this context, we focused on *L. paracasei* WIS43, a novel strain isolated from the breast milk of a healthy volunteer. To address the limited research and pronounced strain-specific differences in UC treatment, we first performed a meta-analysis of publicly available microbiome sequencing datasets from UC patients to identify disease-associated dysbiosis as a theoretical basis for targeted probiotic selection. We then systematically evaluated the protective effects of WIS43 in a DSS-induced mouse colitis model, using both mesalazine and the commercial probiotic strain *L. paracasei* Lpc-37 as controls. Through analyses of inflammatory cytokines, histopathology, 16S rRNA sequencing, and microbial community profiling, we investigated the potential mechanisms by which WIS43 alleviates colitis. We anticipate that this study will expand the functional probiotic repertoire of *Lactobacillus paracasei*, provide additional candidate strains for UC management, and lay the groundwork for the future development of more precise probiotic therapeutic strategies.

## 2 Materials and methods

### 2.1 Meta-analysis of gut microbiota in UC patients

To systematically retrieve gut microbiota data from patients with UC, we conducted literature searches in PubMed and Web of Science using the keywords “ulcerative colitis,” “inflammatory bowel disease,” and “gut microbiota.” We included only English-language original studies that provided publicly available raw sequencing data of the gut microbiota.

FASTQ files of raw sequencing data were downloaded under a Linux environment. Each dataset underwent quality control, denoising, and taxonomic annotation, followed by the generation of an operational taxonomic unit (OTU) table for subsequent community structure analyses.

### 2.2 Bacterial culture

*Lactobacillus paracasei* WIS43 (CGMCC No. 29245) was isolated from human breast milk and provided by Wisbiom (Beijing) Biotechnology Co., Ltd. and Microbiome-Gut-Brain Laboratory (MGBlab) (Beijing, China). The strain was streaked on de Man–Rogosa–Sharpe (MRS) agar plates (Solarbio, M8540) and incubated at 37 °C for ~24 h under anaerobic conditions (85% N₂, 10% CO₂, 5% H₂) in a microaerophilic anaerobic workstation (AMW1000, Hariolab). Single colonies were transferred into liquid MRS broth supplemented with 0.5 g/L L-cysteine hydrochloride and incubated anaerobically at 37 °C for 18 h. The bacterial concentration was determined by colony-forming unit (CFU) counts on MRS agar plates containing 1.5% agar (Saeedi et al., 2020). The reference strain, *L. paracasei* Lpc-37, was obtained from Danisco USA (United States, Madison Plant).

### 2.3 Growth curve determination of *Lactobacillus paracasei* WIS43

Frozen glycerol stock of *Lactobacillus paracasei* WIS43 was revived by inoculating 1% (v/v) into MRS broth and incubating anaerobically at 37 °C for 16 h. A 1% (v/v) aliquot of this overnight culture was then transferred into fresh MRS broth in a 96-well microtiter plate (200 μL per well) and incubated anaerobically at 37 °C for 8 h to obtain a mid-log phase seed culture. Subsequently, 2 μL (1% v/v) of the seed culture was inoculated into 198 μL fresh MRS broth in a new 96-well microtiter plate (final volume 200 μL/well; *n* = 3 technical replicates). Optical density at 600 nm (OD_600_) was automatically recorded every 1 h for 24 h. Blank wells containing uninoculated MRS broth were included for background subtraction. Growth curves were plotted as mean OD_600_ ± SD versus time (h).

### 2.4 Antioxidant activity assays

*L. paracasei* WIS43 and the reference strain *L. paracasei* Lpc-37 were subcultured for three generations. The fermentation broth was then centrifuged, and both the supernatant and the disrupted bacterial suspension were collected. Antioxidant activities were evaluated by measuring DPPH radical-scavenging capacity, hydroxyl radical-scavenging capacity, and total antioxidant capacity.

DPPH radical-scavenging activity was determined using a commercial DPPH assay kit (Nanjing Jiancheng Bioengineering Institute, catalog No. A153-1-1). Hydroxyl radical-scavenging activity was measured using a dedicated hydroxyl radical assay kit (microplate method; Nanjing Jiancheng Bioengineering Institute, catalog No. YX-W-A505). Total antioxidant capacity was assessed using the ferric-reducing antioxidant power microplate assay kit (Nanjing Jiancheng Bioengineering Institute, catalog No. A015-3-1).

### 2.5 Animal experiments

Male KM mice (6 weeks old) were purchased from Beijing HFK Bio-Technology Co., Ltd. (Beijing, China). Animals were housed under specific pathogen-free (SPF) conditions at Tianjin University of Traditional Chinese Medicine, with controlled temperature (24 ± 1 °C), relative humidity (50–70%), and a 12 h light/dark cycle. All procedures were conducted in accordance with the institutional guidelines and approved by the Ethics Committee of Tianjin University of Traditional Chinese Medicine (Approval Number: TCM-LAEC2024209F1368).

A total of 50 mice were randomly divided into five groups (*n* = 10/group): normal control (NC), DSS model, mesalazine (positive drug control), *L. paracasei* Lpc-37 (probiotic control), and *L. paracasei* WIS43. Except for the NC group, all mice received 3% (w/v) DSS (36,000–50,000 Da)in drinking water to induce colitis. Interventions with mesalazine, Lpc-37, or WIS43 were administered for 10 days, while the normal and DSS model groups received 0.2 mL saline per mouse per day. Mesalazine was administered at a dosage of 600 mg/kg/day. Probiotic suspensions were administered by oral gavage at 2 × 10^9^ CFU/mL, 0.2 mL per mouse per day.

At the end of the experiment, mice were anesthetized with 2% pentobarbital sodium (40 mg/kg, i.p.; Sigma-Aldrich, United States). Blood samples were collected by enucleation of the eyeball. Colonic tissues were rinsed with PBS (Solarbio, China) and either fixed in 4% paraformaldehyde (Solarbio, China) or snap-frozen at −80 °C. Cecal tissues and contents were also collected and stored at −80 °C for subsequent 16S rRNA sequencing.

### 2.6 Disease activity index

The severity of colitis was evaluated using the disease activity index (DAI), which integrates weight loss, stool consistency, and presence of gross blood. DAI was calculated as: DAI = (weight loss score + stool consistency score + fecal blood score)/3 (Zou et al., 2024). Scores ranged from 0 to 4, as summarized in Table 1.

**Table 1:** Criteria for DAI scoring.

Score	Weight loss	Stool consistency	Gross blood
0	0%	Normal	None
1	1–5%	Slightly loose	Negative
2	6–10%	Semi-formed loose	Occult blood
3	11–15%	Mucus-like stool	Hematochezia + occult blood
4	>15%	Watery stool	Visible bleeding

### 2.7 Histopathological analysis

Colonic tissues were fixed in 4% paraformaldehyde (Solarbio, China), dehydrated through graded ethanol (70, 80, 95, 100%), cleared in xylene, and embedded in paraffin. Tissue blocks were sectioned into 4–6 μm slices, deparaffinized, rehydrated, and stained with hematoxylin and eosin (H&E). Sections were evaluated under a light microscope, and histopathological changes were scored on a semi-quantitative scale: 0 (normal), 1 (mild), 2 (moderate), 3 (severe), and 4 (very severe).

### 2.8 Quantification of inflammatory cytokines

Blood samples were centrifuged at 4000 rpm for 10 min at 4 °C to obtain serum. Colonic tissues were homogenized in ice-cold PBS (1:9, w/v), centrifuged at 12,000 × g for 15 min, and the supernatant was stored at −80 °C. Protein concentrations were measured using the BCA method. Levels of TNF-*α* (Solarbio, SEKM-0034), IL-1*β* (Solarbio, SEKM-0002), and IL-6 (Solarbio, SEKM-0007) in serum and colonic tissue homogenates were determined using enzyme-linked immunosorbent assay (ELISA) kits, according to the manufacturers’ instructions.

### 2.9 Full-length 16S rRNA sequencing and phylogenetic identification

Fecal DNA was extracted following established protocols. The full-length 16S rRNA gene (V1–V9 regions) was amplified using primers 27F (5′- AGAGTTTGATCCTGGCTCAG −3′) and 1492R (5′- TACGGCTACCTTGTACGACTT −3′). Amplicons were recovered from 2% agarose gels and purified to construct SMRTbell libraries (Pacific Biosciences). Sequencing was performed on a PacBio Sequel II platform by Shanghai Biozeron Biotechnology Co., Ltd. (Shanghai, China). Raw FASTA reads underwent quality filtering and alignment. OTUs were clustered at 97% similarity. Downstream analyses, including relative abundance, α-diversity, and β-diversity, were conducted using R software (version 4.2.3).

The 16S rRNA gene sequence of *L. paracasei* WIS43 was aligned with reference sequences retrieved from NCBI using ClustalW. Phylogenetic relationships were inferred using the Neighbor-Joining (NJ) method (Saitou and Nei, 1987), and node support was assessed with 1,000 bootstrap replicates (Felsenstein, 1985). Evolutionary distances were calculated using the Maximum Composite Likelihood (MCL) model (Tamura et al., 2004), expressed as the number of base substitutions per site. Codon positions included were 1st, 2nd, 3rd, and non-coding regions. All positions containing gaps and missing data were removed, resulting in 1,376 positions in the final dataset. A total of 16 representative *Lactobacillus*/*Lacticaseibacillus* sequences were included in the analysis. All phylogenetic analyses were performed using MEGA7 (Kumar et al., 2016).

### 2.10 Statistical analysis

All statistical analyses were performed using GraphPad Prism 9.5.1 (GraphPad, San Diego, CA, United States). Data are expressed as mean ± standard error of the mean (SEM). One-way analysis of variance (ANOVA) was used for group comparisons. A *p*-value < 0.05 was considered statistically significant.

Bioinformatics and statistical analyses were performed using R software (Version 4.3.2). Differences inα-diversity indices were evaluated using the Wilcoxon rank-sum test. Significant differences in microbial community structure (*β*-diversity) were assessed by Permutational Multivariate Analysis of Variance (PERMANOVA) based on Bray–Curtis distances using the vegan package. To identify differentially abundant taxa (as visualized in volcano plots), the Wilcoxon rank-sum test was employed. The obtained *p*-values were adjusted for multiple comparisons using the Benjamini-Hochberg method to control the false discovery rate. Correlations between gut microbiota abundance and host phenotypes were determined using Spearman’s rank correlation analysis.

## 3 Results

### 3.1 Significant reduction of *Lactobacillus* in UC patients

To investigate the ecological association between gut microbiota composition and UC, we focused on the genus *Lactobacillus* and analyzed its abundance trends in UC patients compared with healthy individuals. Through systematic literature searches, five publicly available studies providing raw gut microbiome sequencing data were included (project IDs: PRJNA398187, PRJEB18471, PRJNA684887, PRJNA866870, and PRJEB33711). All five studies reported relative abundance data at the genus *Lactobacillus* level, with clear group differentiation between UC patients and healthy controls.

**Figure 1:**
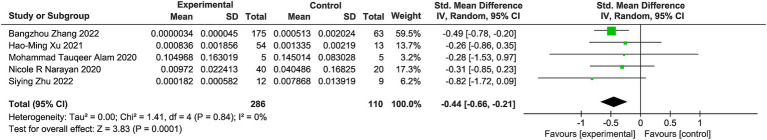
Meta-analysis of the relative abundance of *Lactobacillus* in the gut microbiota of patients with UC compared with healthy controls.

The datasets were obtained from the NCBI SRA database and covered study populations from multiple regions, including Asia and Europe, thereby ensuring both representativeness and diversity. A total of 286 UC patients and 110 healthy controls were included. A meta-analysis was performed using a random-effects model, and the results are presented in Figure 1.

Across all included studies, the relative abundance of *Lactobacillus* was consistently decreased in UC patients. Although confidence intervals overlapped in some individual studies, the effect direction remained consistent. The pooled analysis revealed that *Lactobacillus* abundance was significantly lower in UC patients than in healthy controls (standardized mean difference, SMD = −0.44; 95% CI: −0.66 to −0.21; *p* = 0.0001). Tests of heterogeneity indicated good consistency among studies (I^2^ = 0%, *p* = 0.84), suggesting that the observed effect was stable and reproducible.

In summary, these results support a potential protective role of *Lactobacillus* in maintaining gut homeostasis and counteracting intestinal inflammation. The significant reduction of *Lactobacillus* in UC patients may be closely associated with impaired barrier function and upregulation of pro-inflammatory mediators, highlighting the decrease of this genus as a noteworthy microbial signature in UC pathogenesis.

### 3.2 Biological properties of *Lactobacillus paracasei* WIS43

**Figure 2:**
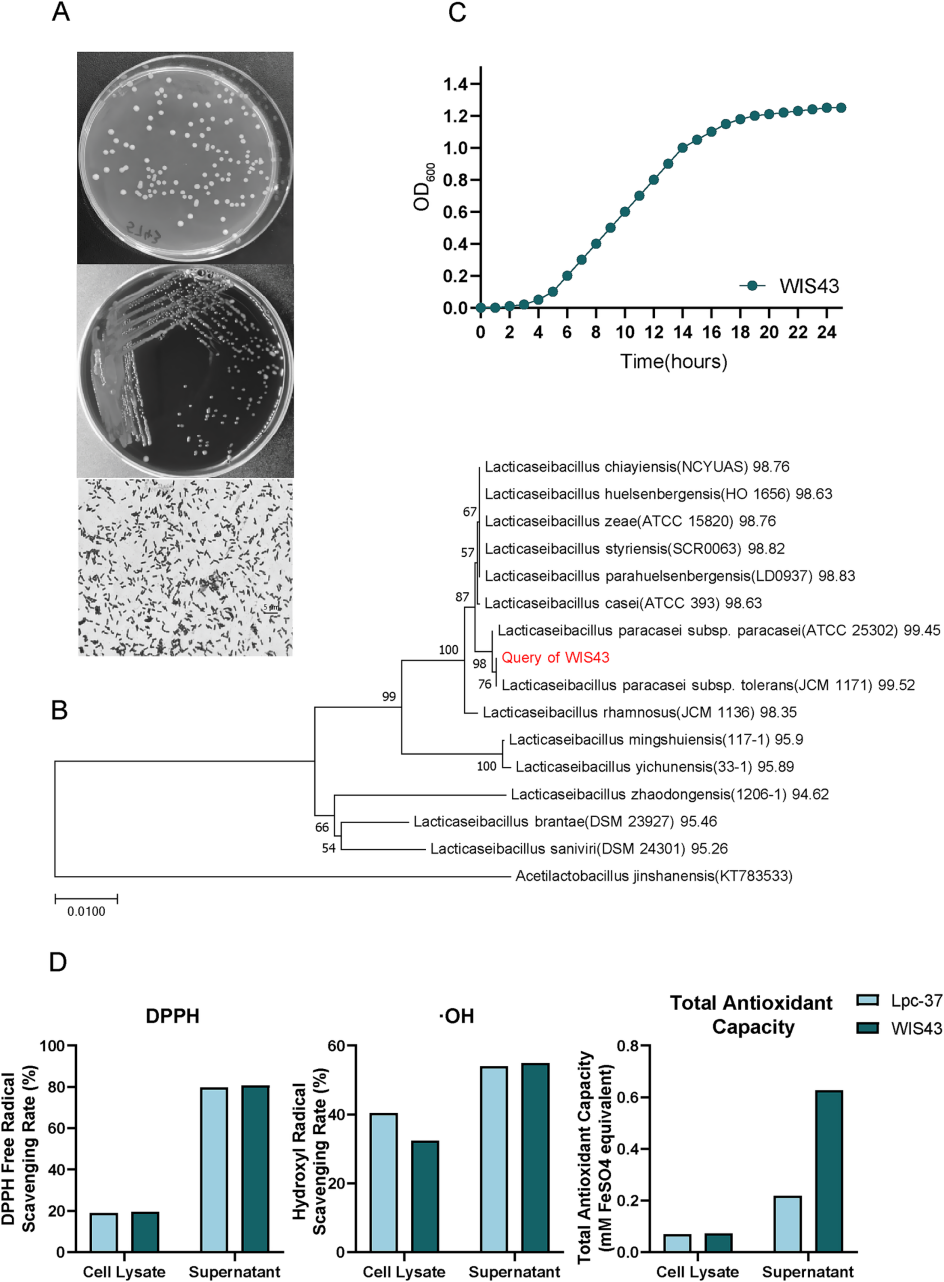
Phenotypic, phylogenetic, growth, and antioxidant characteristics of *L. paracasei* WIS43. **(A)** Colony morphology and Gram staining of WIS43. Top: Colonies on MRS agar after 48 h of anaerobic incubation at 37 °C are white, circular, and convex with smooth surfaces. Middle: Colonies on Columbia blood agar showing no hemolysis. Bottom: Gram staining reveals Gram-positive, short rod-shaped cells. **(B)** Phylogenetic tree based on 16S rRNA gene sequences, showing the evolutionary relationship of WIS43 with other *Lacticaseibacillus* species. The tree was constructed using the neighbor-joining method with 1,000 bootstrap replicates. **(C)** Growth curve of *L. paracasei* WIS43 cultured in MRS broth at 37 °C, with optical density (OD_600_) measured over 24 h. **(D)** Antioxidant activity of WIS43 compared to the reference probiotic *L. paracasei* Lpc-37. The graphs show the scavenging rate of DPPH free radicals (left) and hydroxyl radicals (OH, middle), and the total antioxidant capacity (right) for both the cell lysate and cell-free supernatant.

The *L. paracasei* WIS43 strain was isolated from human breast milk. On MRS agar, WIS43 formed white, circular, and convex colonies with smooth surfaces and entire margins, measuring 1–2 mm in diameter after 48 h of anaerobic incubation at 37 °C, On Columbia blood agar, the colonies appeared semi-transparent and showed no hemolysis (gamma-hemolysis). Microscopic examination following Gram staining revealed that the cells were Gram-positive, non-motile, non-spore-forming short rods (Figure 2A). To further substantiate the taxonomic identity of WIS43, we performed a phylogenetic analysis based on full-length 16S rRNA gene sequences from closely related *Lacticaseibacillus* species. The neighbor-joining tree demonstrated that WIS43 clustered robustly with *L. paracasei* lineages, forming a well-supported clade with *L. paracasei subsp. paracasei* ATCC 25302 and *L. paracasei subsp. tolerans* JCM 1171, with bootstrap values exceeding 90% (Figure 2B). Evolutionary distances calculated using the Maximum Composite Likelihood (MCL) model showed minimal divergence within this clade, confirming that WIS43 falls within the *L. paracasei* species boundary and is taxonomically distinct from related species such as *L. casei*, *L. rhamnosus*, and *L. zeae*. The combination of phenotypic profiling, carbohydrate utilization patterns, and robust phylogenetic placement provides strong taxonomic evidence for classifying WIS43 as a novel *L. paracasei* strain. Phenotypic profiling using the API 50 CHL system demonstrated that WIS43 ferments a broad spectrum of carbohydrates—including ribose, galactose, glucose, fructose, mannose, and N-acetylglucosamine—while remaining negative for substrates such as glycerol, erythritol, D-arabinose, L-arabinose, D-xylose, and adonitol (Supplementary Table S1).

The growth kinetics of WIS43 in MRS broth further revealed a brief lag phase (approximately 5 h) before entering the logarithmic growth phase. The strain subsequently reached the stationary phase at approximately 20 h, achieving a maximum OD_600_ of approximately 1.2 (Figure 2C). Furthermore, WIS43 also displayed superior gastrointestinal resilience. In tolerance assays simulating gastric and intestinal fluids, both *L. paracasei* strains maintained survival rates above 50%. However, WIS43 consistently exhibited significantly higher viability than the commercial strain Lpc-37, demonstrating its enhanced tolerance to adverse gastrointestinal environments (Supplementary Figure S1).

Beyond survival in challenging environments, the functional potential of WIS43 was also investigated. Notably, WIS43 demonstrated markedly enhanced antioxidant capacity compared with Lpc-37. Both the cell-free supernatant and the cell lysate showed significantly higher free radical–scavenging activities. The supernatant exhibited a DPPH radical–scavenging rate of 79.76%, a hydroxyl radical–scavenging rate of 54.87%, and a total reducing power equivalent to 0.6266 mM FeSO₄, highlighting its strong redox-modulatory potential (Figure 2D; Supplementary Table S3). Additional antibacterial activity profiles are provided in the Supplementary Table S2.

Collectively, the robust growth properties, broad carbohydrate utilization, exceptional gastrointestinal tolerance, and pronounced antioxidant activity of WIS43 support its designation as a promising next-generation probiotic candidate for intestinal health applications.

### 3.3 *Lactobacillus paracasei* WIS43 ameliorated pathological symptoms in DSS-induced UC mice

To systematically evaluate the therapeutic potential of *L. paracasei* in UC, we investigated WIS43 in a DSS-induced murine colitis model. Previous studies have shown that different *L. paracasei* strains exert anti-inflammatory effects through distinct mechanisms, such as modulating immune balance (Huang et al., 2021; Su et al., 2024) and enhancing epithelial barrier function (Tarrah et al., 2021). However, strain-specific differences in efficacy remain to be clarified.

**Figure 3:**
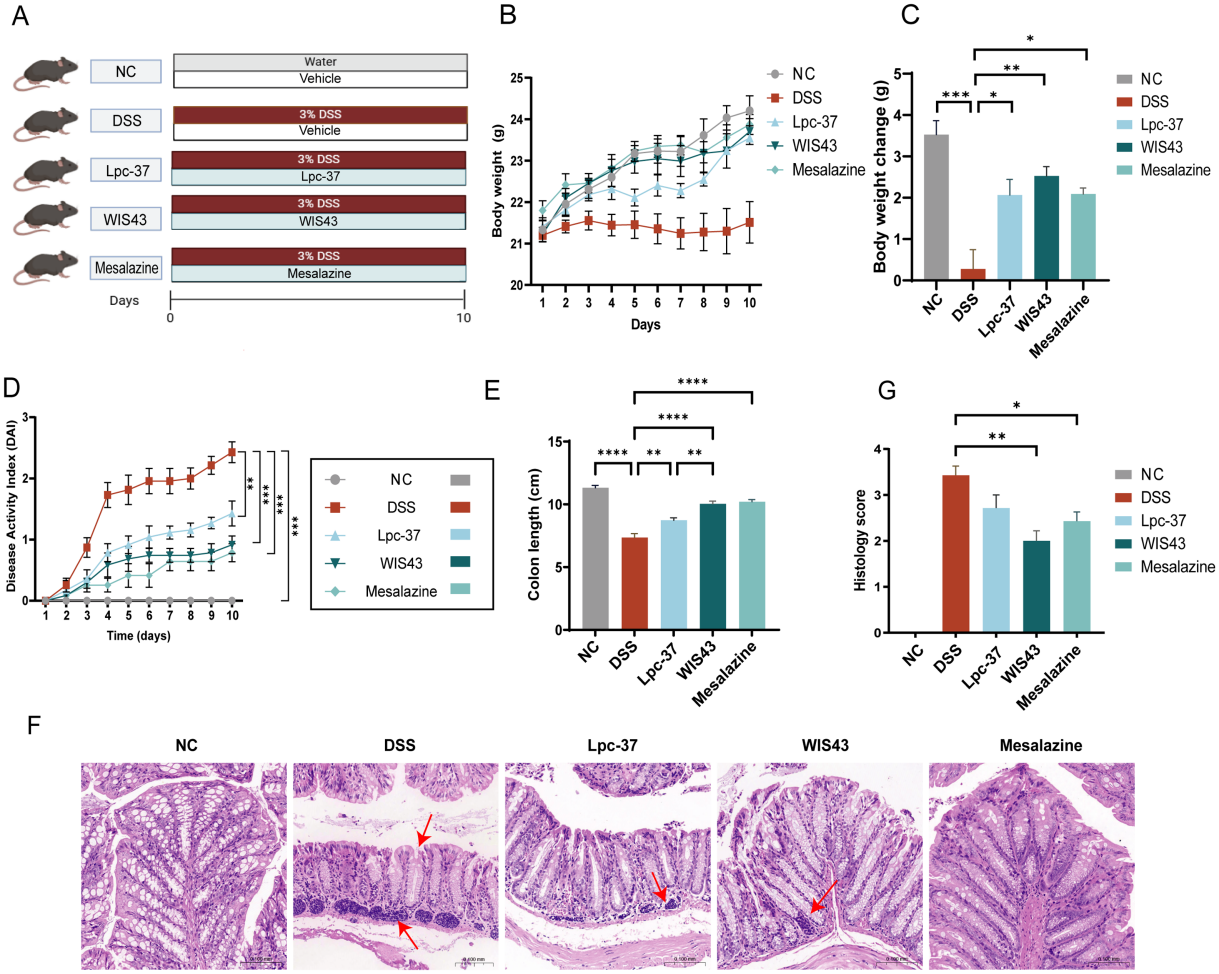
Therapeutic effects of probiotics on DSS-induced colitis in mice. **(A)** Experimental design. Mice received 3% DSS in drinking water for 10 days and were administered daily gavage with *L. paracasei* Lpc-37 (2 × 10^9^ CFU), *L. paracasei* WIS43 (2 × 10^9^ CFU), or mesalazine (positive control). NC, normal control group. **(B)** The weight change curves for each group of mice. **(C)** Body weight changes. DSS caused significant weight loss, which was alleviated by probiotic or mesalazine treatment. WIS43- and mesalazine-treated groups showed body weight recovery comparable to NC. **(D)** Disease activity index (DAI) over 10 days. DSS induced a rapid and sustained increase in DAI, while probiotics and mesalazine significantly attenuated disease severity. **(E)** Colon length measurements. DSS significantly shortened colon length, whereas treatment with probiotics or mesalazine preserved colon length. WIS43 restored colon length to levels comparable with NC. **(F)** Representative H&E-stained sections of colonic tissues from each group (scale bar = 100 μm). WIS43 treatment preserved epithelial and crypt morphology with minimal inflammatory infiltration, resembling NC. **(G)** Histopathological scores. Probiotic and mesalazine treatments significantly reduced epithelial injury, crypt damage, and inflammatory infiltration. Data are expressed as mean ± SEM (*n* = 10 per group). *p < 0.05, **p < 0.01, ****p < 0.0001 vs. DSS (one-way ANOVA).

In our model, mice received 3% DSS in drinking water for 10 consecutive days (Figure 3A). NC mice showed steady body weight gain throughout the experiment, reaching a final average weight of 24.20 ± 1.14 g, whereas DSS-treated mice exhibited growth arrest and declined to 22.90 ± 0.72 g by Day 10. In contrast, probiotic or mesalazine intervention alleviated DSS-induced weight loss. As shown in Figures 3B,C, Lpc-37, WIS43, and mesalazine all promoted significant weight recovery, with WIS43 achieving slightly greater improvement than Lpc-37 and mesalazine. These results indicate that WIS43, similar to mesalazine, effectively prevented DSS-induced weight loss.

The DAI, which integrates body weight loss, stool consistency, and rectal bleeding, was also evaluated (Figure 3D). DAI scores ranked from highest to lowest as DSS group > Lpc-37 group > WIS43 group ≈ mesalazine group > normal group. DSS mice exhibited a sharp increase in DAI from Day 3 onward, which remained elevated throughout the experiment. Administration of mesalazine, Lpc-37, or WIS43 significantly reduced DAI scores compared with DSS controls. Notably, WIS43 demonstrated greater efficacy than Lpc-37, with DAI values approaching those of the mesalazine group after Day 7.

Colon length was assessed as an indicator of structural damage (Figure 3E). DSS administration markedly shortened colon length compared with the NC group (*p* < 0.0001). Treatment with mesalazine, Lpc-37, or WIS43 significantly restored colon length compared with DSS controls (*p* < 0.01). Among them, WIS43 treatment resulted in a significantly greater improvement than Lpc-37 and achieved colon lengths comparable to those of the mesalazine group (*p* > 0.05), indicating a protective efficacy comparable to mesalazine and superior to Lpc-37.

Histological analysis further supported these findings (Figure 3F). NC group exhibited intact epithelium, preserved goblet cells, and well-defined crypts without inflammatory infiltration. DSS-treated mice displayed severe epithelial injury, crypt distortion and loss, and prominent inflammatory infiltration. Mesalazine-treated mice showed largely preserved epithelium with minimal infiltration. Lpc-37 partially improved tissue architecture but residual inflammatory infiltration persisted. In contrast, WIS43 treatment preserved epithelial and crypt morphology, with minimal inflammatory infiltration, closely resembling the normal group. Histopathological scores (Figure 3G) were significantly reduced by mesalazine and WIS43 compared with DSS controls (*p* < 0.05), whereas Lpc-37 did not reach statistical significance (*p* > 0.05). Importantly, WIS43 showed lower injury scores than Lpc-37, comparable to mesalazine.

Overall, these results demonstrate that *L. paracasei* WIS43 effectively alleviates clinical symptoms, preserves colon structure, and reduces histological injury in DSS-induced colitis, supporting its potential as a promising non-pharmacological strategy for UC management.

### 3.4 *Lactobacillus paracasei* WIS43 reduced inflammatory cytokine levels in DSS-induced UC mice

Inflammatory cytokines are markedly elevated in DSS-induced colitis. To assess the anti-inflammatory effects of *L. paracasei* WIS43, levels of TNF-*α*, IL-1β, and IL-6 were measured in both serum and colonic tissues.

**Figure 4:**
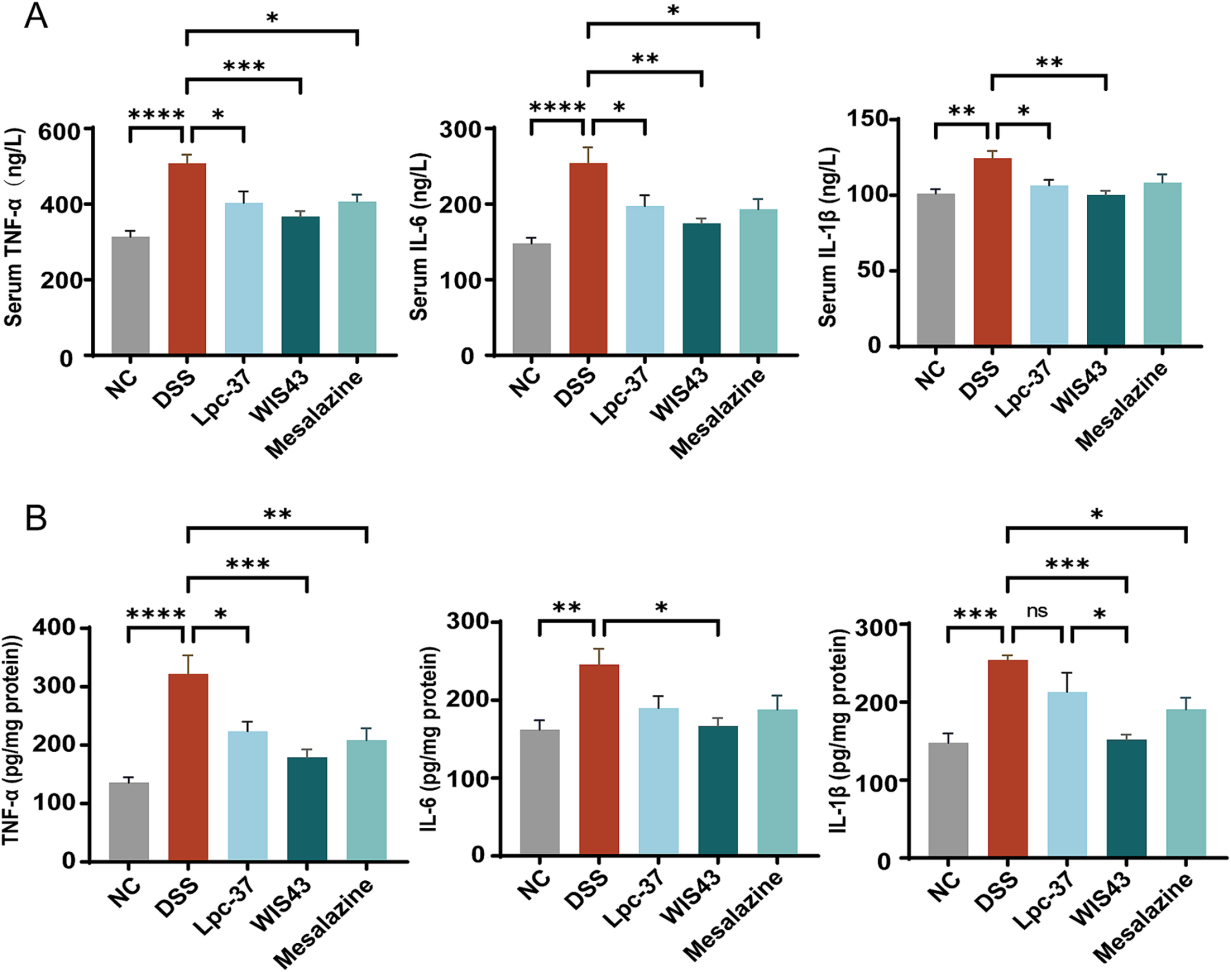
Anti-inflammatory effects of probiotics in DSS-induced colitis mice. **(A)** Serum cytokine levels (TNF-α, IL-6, and IL-1β). DSS treatment significantly increased cytokine concentrations compared with the NC group. Lpc-37 reduced TNF-α and IL-1β but had no effect on IL-6, whereas WIS43 and mesalazine significantly decreased all three cytokines. **(B)** Colonic cytokine levels. DSS markedly elevated TNF-α, IL-6, and IL-1β compared with NC. Lpc-37 reduced TNF-α only, whereas WIS43 significantly decreased all three cytokines, showing an effect comparable to mesalazine (*n* = 10 per group). *p < 0.05, **p < 0.01, ***p < 0.001, ****p < 0.0001 vs. DSS.

As shown in Figure 4A, serum concentrations of TNF-α, IL-6, and IL-1β were markedly elevated in DSS-treated mice compared with NC (*p* < 0.0001, *p* < 0.01). Lpc-37 intervention significantly reduced TNF-α and IL-1β levels (*p* < 0.05), but did not affect IL-6 (*p* > 0.05). In contrast, both WIS43 and mesalazine significantly decreased all three cytokines, with reductions in TNF-α (*p* < 0.001), IL-6 (*p* < 0.01), and IL-1β (*p* < 0.01), demonstrating broader anti-inflammatory effects.

Consistent results were observed in colonic tissues (Figure 4B). DSS markedly upregulated TNF-α, IL-1β, and IL-6, compared with NC (*p* < 0.001, *p* < 0.01). WIS43 treatment significantly reduced all three cytokines (*p* < 0.05), whereas Lpc-37 decreased only TNF-α (*p* < 0.05). Notably, the suppression of inflammatory mediators by WIS43 was more pronounced than that of Lpc-37 and comparable to mesalazine, underscoring the potent anti-inflammatory activity of WIS43.

### 3.5 *Lactobacillus paracasei* WIS43 improved gut microbiota composition in DSS-induced UC mice

**Figure 5:**
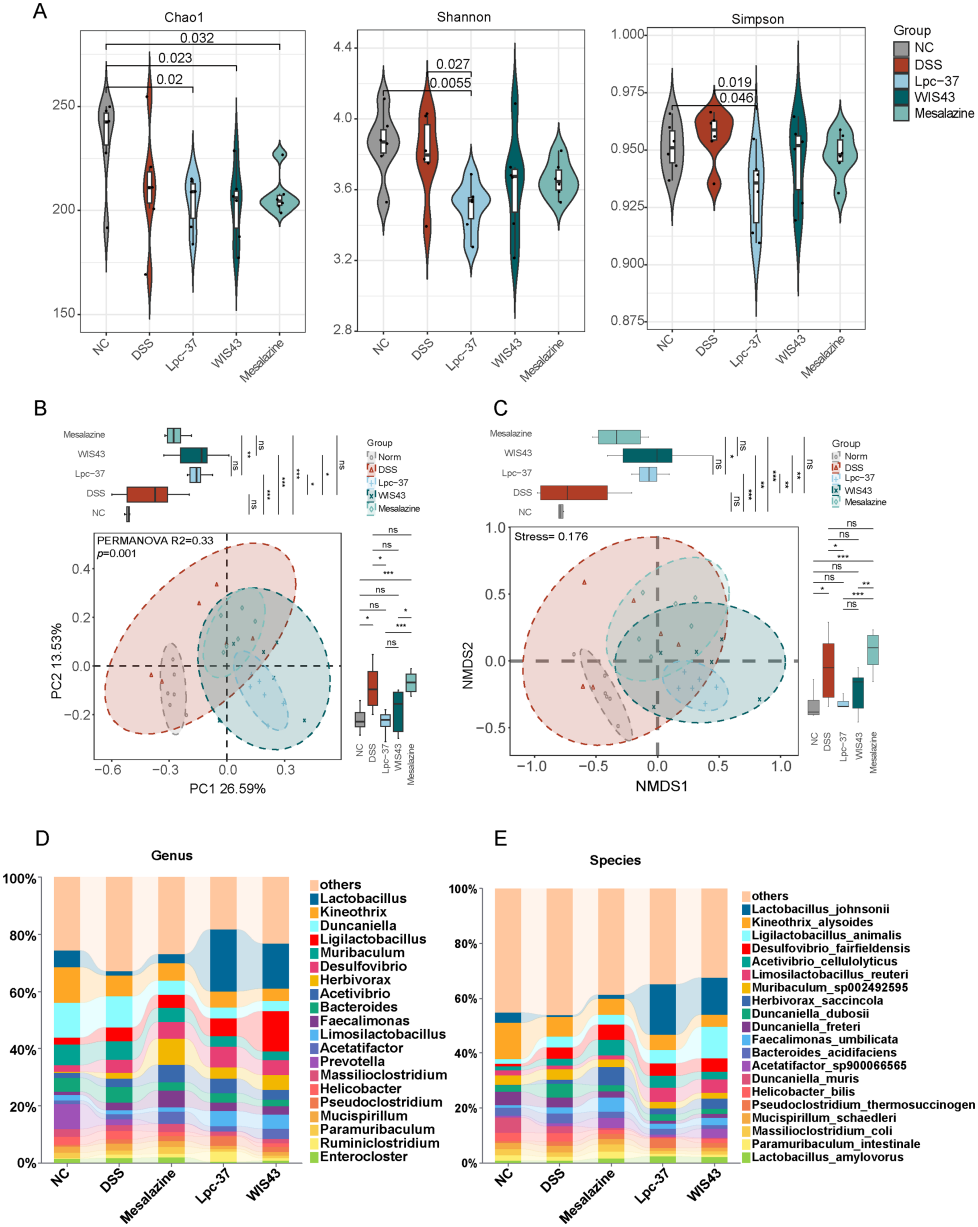
Effects of *Lactobacillus paracasei* interventions on gut microbiota composition in DSS-induced colitis mice. **(A)** Alpha diversity indices (Chao1, Shannon, Simpson). WIS43 significantly increased Shannon and Simpson indices compared with DSS, indicating partial restoration of microbial richness and evenness. **(B)** Principal coordinate analysis (PCoA) based on Bray–Curtis distances revealed distinct clustering of microbial communities among groups (PERMANOVA, *R*^2^ = 0.33, *p* = 0.001). **(C)** Non-metric multidimensional scaling (NMDS) analysis confirmed separation of microbial compositions, with a stress value of 0.176, indicating reliable ordination. For microbiota analysis, *n* = 6 samples were randomly selected from each group. **(D,E)** Relative abundance of gut microbiota at the genus **(D)** and species **(E)** levels. WIS43 restored the abundance of beneficial taxa such as *L. johnsonii* and *L. animalis* while reducing potentially pathogenic taxa including *Kineothrix* and *Duncaniella*.

To evaluate the effects of *L. paracasei* WIS43 on the gut microbiota of DSS-induced colitis mice, 16S rRNA gene sequencing was performed. Analysis of α diversity revealed that compared to the control group, the DSS-treated group showed an upward trend in the Simpson index, while Shannon and Chao1 indices exhibited downward trends, though no significant differences were observed (Figure 5A). Compared to the control group, both Lpc-37 and WIS43 strains further reduced Chao1, Shannon, and Simpson diversity indices to varying degrees. Mesalazine had no significant effect on α diversity.

In contrast, Principal Coordinate Analysis (PCoA) and Non-metric Multidimensional Scaling (NMDS) clearly demonstrated microbial community restructuring. In the PCoA analysis (Figure 5B), the DSS-treated group formed distinct clusters separated from the normal group along the PC2 axis (*p* < 0.05), while the WIS43 and Lpc-37 groups exhibited significant separation from the DSS group along the PC1 dimension (*p* < 0.05). These findings were corroborated by NMDS analysis (Figure 5C), where both Lpc-37 and WIS43 showed significant separation from the DSS group along the NMDS1 dimension (*p* < 0.01). Additionally, the Lpc-37 group exhibited significant separation from the DSS group along the NMDS2 dimension (*p* < 0.05). At the genus level (Figure 5D), *Lactobacillus*, *Kineothrix*, and *Duncaniella* were dominant taxa. Compared with the normal group, *Lactobacillus* abundance was markedly decreased in the DSS group. Both WIS43 and Lpc-37 significantly increased the relative abundance of *Lactobacillus* while reducing *Kineothrix* and *Duncaniella*. At the species level (Figure 5E), WIS43 and Lpc-37 significantly enriched *Lactobacillus johnsonii* and *Ligilactobacillus animalis*, taxa previously associated with anti-inflammatory and barrier-protective functions (Boll et al., 2024; Liu et al., 2025).

Together, these findings suggest that WIS43 supplementation reshapes gut microbiota composition, characterized by increased beneficial lactobacilli and decreased pro-inflammatory taxa, thereby contributing to its protective effects against colitis.

**Figure 6:**
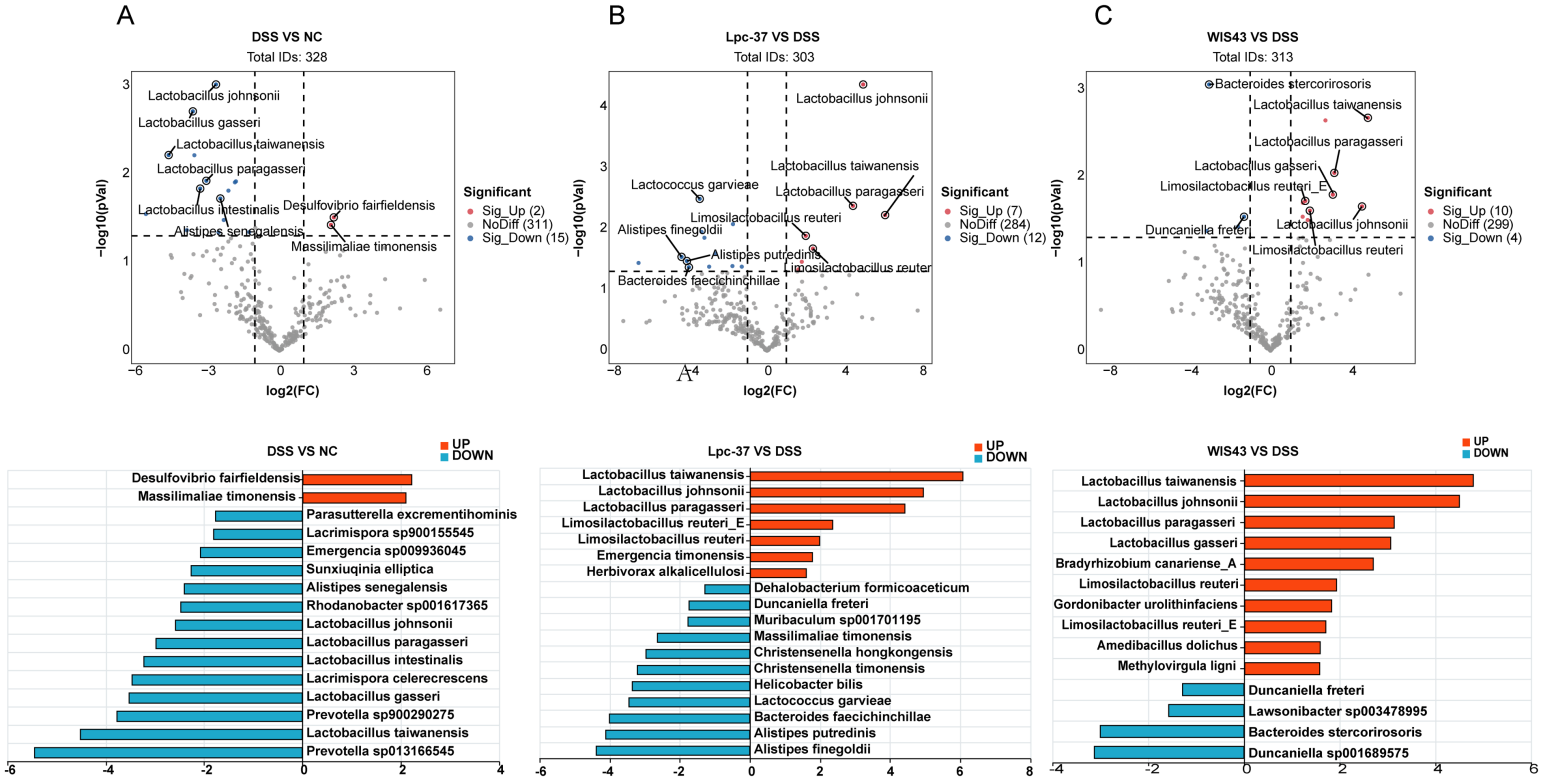
Strain-specific modulation of gut microbial species in DSS-induced colitis mice. **(A–C)** Volcano plot analysis of differential microbial species (|log₂FC| > 1, *p* < 0.05). **(A)** DSS vs. NC. Several *Lactobacillus* species (*L. johnsonii*, *L. paragasseri*, *L. intestinalis*, *L. gasseri*, *L. taiwanensis*) were significantly decreased, while *D. fairfieldensis* and *M. timonensis* were enriched. **(B)** Lpc-37 vs. DSS. Lpc-37 intervention increased beneficial taxa including *L. johnsonii*, *L. paragasseri*, *L. taiwanensis*, and *L. reuteri*, while reducing harmful taxa such as *Alistipes* spp. and *Bacteroides faecichinchillae*. **(C)** WIS43 vs. DSS. WIS43 significantly enriched *L. johnsonii*, *L. paragasseri*, *L. taiwanensis*, *L. gasseri*, and *L. reuteri*, while decreasing *D. freteri*, *Lawsonibacter* sp003478995, and *B. stercorirosoris*.

To further assess the significance and biological relevance of microbiota alterations, volcano plot analysis was performed, integrating fold change (FC) and statistical significance. As shown in Figure 6, DSS treatment induced marked changes in microbial composition compared with the normal group. Several beneficial *Lactobacillus* species, including *L. johnsonii*, *L. paragasseri*, *L. intestinalis*, *L. gasseri*, and *L. taiwanensis*, were significantly decreased, whereas only a few taxa, such as *Desulfovibrio fairfieldensis* and *Massilimaliae timonensis*, were enriched.

Compared with DSS mice, the Lpc-37 group exhibited significant increases in *L. taiwanensis*, *L. johnsonii*, *L. paragasseri*, *Limosilactobacillus reuteri_E*, and *L. reuteri*, along with reductions in potentially harmful taxa such as *Alistipes putredinis*, *Alistipes finegoldii*, and *Bacteroides faecichinchillae*. Similarly, WIS43 supplementation significantly enriched *L. taiwanensis*, *L. johnsonii*, *L. paragasseri*, *L. gasseri*, and *L. reuteri*, while decreasing the abundance of *Duncaniella freteri*, *Lawsonibacter* sp003478995, and *Bacteroides stercorirosoris*.

Taken together, these results indicate that WIS43 markedly improved the gut microbiota composition of DSS-treated mice. Notably, the relative abundance of beneficial taxa, particularly members of the *Lactobacillus* genus, was significantly increased in the WIS43 group, effectively counteracting the DSS-induced depletion of *Lactobacillus*. This suggests that WIS43 exerts its anti-inflammatory effects, at least in part, by modulating the gut microbiota toward a more favorable, probiotic-enriched profile.

### 3.6 Spearman correlation analysis between gut microbiota and colitis-related parameters

**Figure 7:**
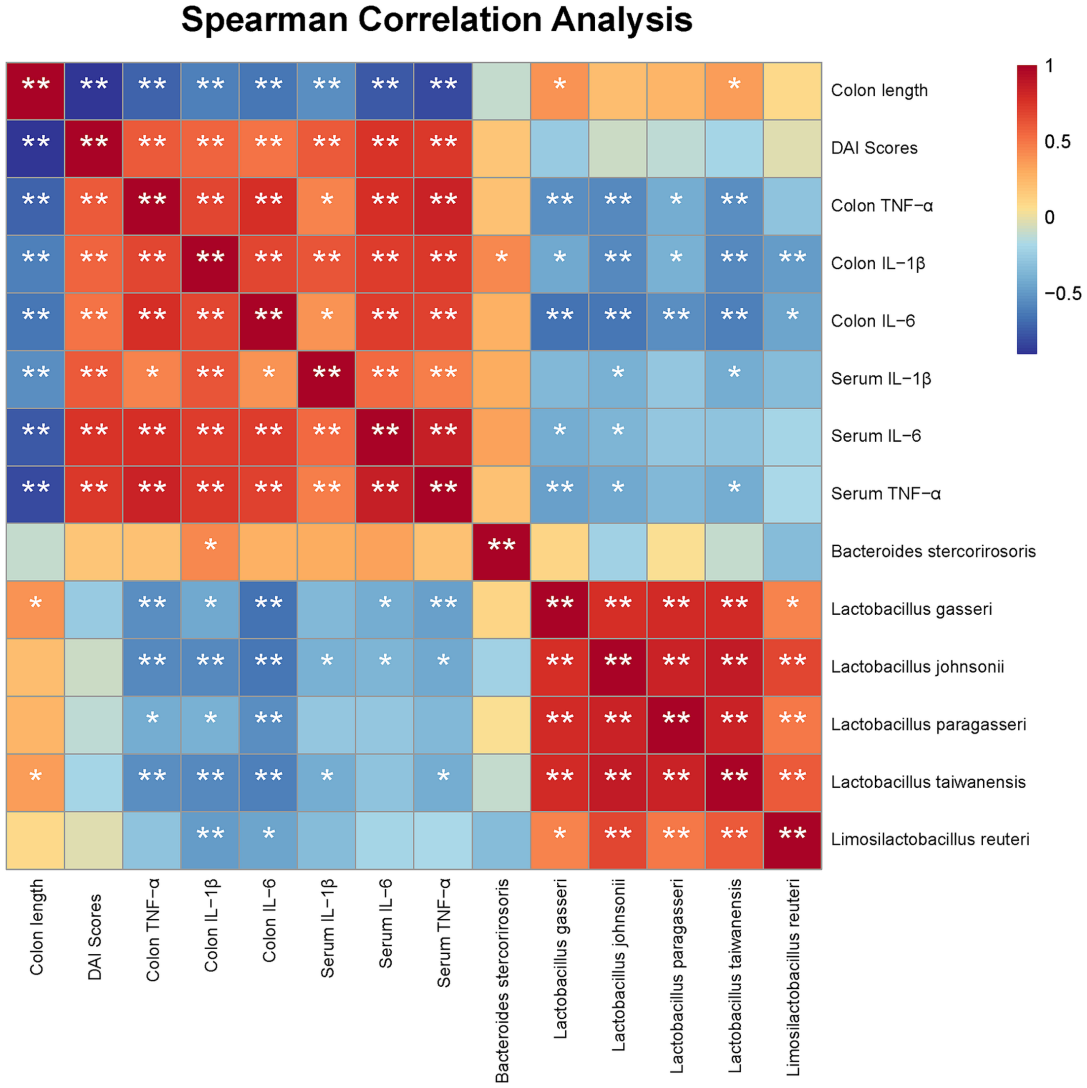
Spearman correlation heatmap between gut microbiota and colitis-related phenotypic and inflammatory parameters. Heatmap showing correlations between colon length, DAI scores, inflammatory cytokines (colonic and serum TNF-α, IL-1β, IL-6), and the relative abundance of key bacterial taxa. Blue indicates negative correlations, red indicates positive correlations. *p < 0.05, **p < 0.01 (Spearman correlation).

To further explore the potential associations between specific microbial taxa and colitis-related phenotypic indicators, Spearman correlation analysis was performed, and a heatmap was constructed to visualize correlations between bacterial abundance and inflammatory parameters (Figure 7). Distinct correlation patterns were observed between lactic acid bacteria and indices of intestinal inflammation.

At the host phenotype level, colon length was strongly negatively correlated with disease activity index (DAI) scores (r < −0.6, *p* < 0.01), as well as with colonic and serum levels of IL-1β, IL-6, and TNF-*α*, indicating that shorter colons were associated with higher inflammatory status and more severe disease. In contrast, DAI scores were positively correlated with all inflammatory cytokines (r > 0.6, *p* < 0.01), confirming their validity as indicators of disease activity.

At the microbial level, Spearman correlation analysis (Figure 7) revealed that specific *Lactobacillus* species enriched by WIS43 intervention were significantly associated with key inflammatory markers. Specifically, the relative abundances of *L. gasseri*, *L. johnsonii*, *L. paragasseri* and *L. taiwanensis* showed significant negative correlations with colonic levels of TNF-α, IL-1β, and IL-6 (*p* < 0.05 or *p* < 0.01). Furthermore, *L. gasseri* and *L. taiwanensis* exhibited significant positive correlations with colon length (*p* < 0.05). In contrast, *B. stercorirosoris* was significantly positively correlated with colonic IL-1*β* levels (*p* < 0.05). That suggesting a potential role in promoting inflammation.

Overall, this correlation analysis further supports the notion that the enrichment of beneficial lactobacilli by WIS43 intervention may play a critical role in alleviating colonic inflammation and improving gut health.

## 4 Discussion

The incidence of UC has been steadily increasing worldwide, making it a pressing global public health challenge (Ananthakrishnan et al., 2020; Olfatifar et al., 2021). Currently, therapeutic options for UC remain limited, highlighting the urgent need for the development of novel interventions. In this context, probiotics have gained increasing attention as promising candidates for alternative or adjunctive therapies (Zhou et al., 2022).

Probiotics exert multidimensional therapeutic effects in UC, including reinforcement of the intestinal barrier (Wang et al., 2025; Zhao et al., 2025), modulation of gut microbiota structure and immune homeostasis (Żak-Bochenek et al., 2024), and enhancement of metabolic activity (Peña-Durán et al., 2025). A healthy intestinal mucosal barrier is essential for maintaining homeostasis and defending against external pathogens. Several studies have demonstrated that probiotics can strengthen barrier function through diverse molecular mechanisms. For example, *L. reuteri* was reported to promote *Bacteroides acidifaciens*-mediated pentadecanoic acid synthesis, thereby upregulating tight junction proteins (ZO-1, occludin) and inhibiting NF-κB activation, which collectively reduced intestinal permeability and inflammatory responses (Wu et al., 2024).

Another important mechanism involves restructuring of gut microbial composition. Probiotic supplementation has been shown to restore microbial diversity. For instance, *Lactobacillus plantarum* HNU082 increased the abundance of multiple beneficial taxa, including *L. plantarum*, *B. pseudolongum*, *Akkermansia muciniphila*, *Parabacteroides distasonis*, and *L. reuteri*, thereby improving *α*-diversity and optimizing β-diversity in the murine cecal microbiota (Wu et al., 2022). Among widely recognized probiotics, lactobacilli and bifidobacteria play particularly prominent roles in promoting intestinal health and alleviating IBD. *Lactobacillus* species, as important members of the mammalian commensal microbiota, are not only broadly distributed in the host but are also widely used in the food industry. Their ability to help maintain intestinal homeostasis underscores their significance in the management of gut health.

Based on the established mechanisms of probiotics, we further evaluated the therapeutic efficacy of *L. paracasei* WIS43 in a DSS-induced murine model of UC. DSS is the most widely used chemical agent for experimental colitis models due to its simplicity, short induction cycle, and reproducibility (Solomon et al., 2010). In our study, administration of 3% DSS in drinking water for 10 days successfully induced colitis symptoms in mice. Consistent with previous reports, both Lpc-37 and WIS43 alleviated DSS-induced weight loss and colon shortening. However, in terms of DAI scores, Lpc-37 was only partially effective, whereas WIS43 performed comparably to mesalazine, with no significant difference between the two groups. Histological analysis further supported these findings: while Lpc-37–treated mice still exhibited residual epithelial damage and inflammatory infiltration, WIS43 markedly preserved epithelial integrity and reduced histopathological scores. These results suggest that, despite belonging to the same species, WIS43 exerts superior therapeutic effects compared with Lpc-37, underscoring its potential as a non-pharmacological strategy for UC.

Histological assessment of colonic tissues is considered the “gold standard” for evaluating UC severity (Münch and Langner, 2015). Using hematoxylin and eosin (H&E) staining, we observed that WIS43 markedly reduced epithelial injury and inflammatory cell infiltration compared with DSS, leading to significantly lower histopathological scores. In contrast, residual epithelial damage and infiltration were still evident in Lpc-37–treated mice. These results indicate that, although both strains belong to *L. paracasei*, WIS43 exhibited superior therapeutic efficacy compared with Lpc-37 and comparable effects to mesalazine, supporting its potential as a non-pharmacological intervention for UC.

UC pathogenesis is multifactorial, with immune dysregulation and cytokine imbalance recognized as central mechanisms (Neurath, 2014). Elevated pro-inflammatory cytokines, including TNF-*α*, IL-1β, and IL-6, are closely associated with UC severity. TNF-α induces apoptosis and inflammatory responses in colonic epithelial cells, thereby disrupting epithelial integrity, increasing intestinal permeability, and aggravating inflammation (Pugliese et al., 2017). IL-1β and IL-6 play critical roles in autoimmune and inflammatory cascades (Tanaka et al., 2014; Nakase et al., 2022). In our study, WIS43 significantly reduced TNF-α, IL-1β, and IL-6 levels in both serum and colonic tissues, whereas Lpc-37 exerted only limited effects, lowering IL-1β in serum and TNF-α in colon tissue. These findings indicate that WIS43 exerts stronger anti-inflammatory activity, likely through suppression of cytokine secretion, which likely represents a key mechanism mediating its protective effect in UC.

With the rapid advancement of microbiome research, the role of gut microbiota in UC has been increasingly emphasized. Numerous studies have demonstrated that UC patients exhibit decreased microbial diversity, reduced beneficial taxa (e.g., *Lactobacillus*, *Alistipes*), and increased harmful taxa (e.g., *Escherichia coli*) (Pittayanon et al., 2020). Our meta-analysis confirmed that the relative abundance of *Lactobacillus* was significantly reduced in UC patients compared with healthy controls (SMD = −0.44; 95% CI: −0.66 to −0.21; *p* = 0.0001; I^2^ = 0%), reinforcing the importance of this genus as a “keystone symbiont” in gut homeostasis. Consistently, DSS treatment markedly decreased *Lactobacillus* abundance in our murine model, whereas WIS43 supplementation restored its levels. This aligns with the growing view that targeting gut microbiota is a rational and feasible approach for UC therapy. Supporting evidence comes from studies using other probiotic strains, such as *Lactobacillus casei* IB1, which alleviated colitis by modulating microbiota composition (increasing *Faecalibaculum* and *Alistipes*, reducing *Bacteroides*) (Lao et al., 2024), and *Lactobacillus acidophilus* 6,074, which promoted the growth of lactobacilli, bifidobacteria, and *Akkermansia* while enhancing SCFA production (Zhao et al., 2025).

Importantly, WIS43 restored the abundance of multiple beneficial *Lactobacillus* species, including *L. taiwanensis*, *L. johnsonii*, *L. gasseri*, and *L. reuteri*. Previous studies have highlighted the protective roles of these taxa in intestinal health. For instance, *L. taiwanensis* was positively associated with enhanced intestinal barrier function in high-fat diet-induced mice (Cai et al., 2025). *L. johnsonii* has been shown to prevent DSS-induced colitis by modulating the gut microbiota–SCFA axis to inhibit M1 macrophage polarization, and by activating the TLR1/2–STAT3 pathway to promote IL-10 production (Jia et al., 2022). *L. gasseri* strengthens epithelial barrier integrity by upregulating E-cadherin expression (Qian et al., 2024), whereas *L. reuteri* not only enhances barrier function (Lin et al., 2023) but also promotes the production of anti-inflammatory metabolites such as pentadecanoic acid (Wu et al., 2024). The enrichment of these taxa in WIS43-treated mice suggests a multifaceted protective mechanism involving restoration of microbial balance, barrier reinforcement, and immune modulation.

Beyond *Lactobacillus* species, WIS43 also modulated several functional non-lactic acid bacteria. *Gordonibacter urolithinfaciens*, a gut bacterium capable of producing active catechol dehydroxylase, metabolizes dietary ellagic acid (a polyphenol abundant in fruits) into urolithin A (UA). UA has been shown to improve cellular health by enhancing mitophagy and mitochondrial function while suppressing deleterious inflammation, thereby exerting antioxidant, anti-aging, and anti-inflammatory effects (D'Amico et al., 2021). It has been implicated in the prevention of colitis (Singh et al., 2019; Ma et al., 2023), osteoarthritis (D'Amico et al., 2022), and Alzheimer’s disease (Hou et al., 2024). Another taxon, *Bacteroides stercorirosoris*, is a common member of the gut microbiota and has been identified as a potential biomarker in non-alcoholic fatty liver disease (NAFLD), with significantly higher abundance in NAFLD patients than in healthy controls (Ni et al., 2023). Interestingly, its abundance is enriched in early-stage but decreased in late-stage hepatocellular carcinoma (Esparteiro et al., 2025). *Lawsonibacter* species, isolated as butyrate producers from human feces (Sakamoto et al., 2018), have also been reported to increase following probiotic supplementation in a *Toxoplasma gondii* model, concomitant with elevated short-chain fatty acid production and anti-inflammatory effects (Lee et al., 2024). In contrast, the response of *Lawsonibacter* to WIS43 intervention in our study differed from previous findings, which may be attributable to differences in animal models, microbial interactions, or host-specific factors. These discrepancies warrant further investigation.

We further evaluated the impact of WIS43-mediated regulation of beneficial and potentially pathogenic bacteria from the perspective of intestinal microbial ecosystem stability. Indiscriminate increases in certain beneficial taxa or excessive depletion of others can create new ecological vacancies or functional redundancies, ultimately disrupting community homeostasis. In the present study, WIS43 significantly enriched well-documented anti-inflammatory species (e.g., *L. johnsonii*, *L. taiwanensis*, *L. gasseri*, and *L. reuteri*) while only modestly reducing the abundance of potential opportunistic pathogens (e.g., *D. freteri* and *B. stercorirosoris*), without causing overdominance of any single taxon (highest relative abundance <15%). Importantly, *α*-diversity (Shannon and Simpson indices) was significantly restored toward levels observed in healthy controls. Spearman correlation analysis further revealed strong negative associations between WIS43-enriched beneficial lactobacilli and clinical/inflammatory markers, whereas suppressed taxa exhibited positive correlations. These findings indicate that WIS43 optimizes the beneficial-to-pathogenic ratio while preserving overall community diversity and stability, supporting its safety and sustainability as a next-generation probiotic. Beyond these taxonomic shifts, the present study also illustrates the methodological advantages of full-length 16S rRNA sequencing over conventional short-read approaches. Most previous UC-related microbiota studies have relied on amplification of partial hypervariable regions (e.g., V3–V4), which often lack sufficient resolution to distinguish closely related *Lactobacillus* species and frequently collapse them into genus-level or ambiguous “*Lactobacillus* spp.” categories. This limits the ability to link specific commensal species to ecological functions or therapeutic responses. In contrast, the full-length 16S strategy used here provided more robust species-level assignments, enabling us to resolve functionally relevant taxa such as *L. taiwanensis, L. johnsonii, L. gasseri, L. reuteri* and *G. urolithinfaciens* in WIS43-treated mice. This higher taxonomic resolution allowed a more precise characterization of the microbial targets of WIS43 and a clearer interpretation of how probiotic intervention reshapes the gut ecosystem in UC.

Compared with previously reported *L. paracasei* strains, WIS43 exhibits a broader and more integrated therapeutic profile in the treatment of ulcerative colitis. Many strains within this species alleviate experimental colitis via distinct single pathways—for example, regulating Th17/Treg balance (strain R3) (Huang et al., 2021), enhancing tight-junction proteins (BNCC345679) (Ahmad et al., 2024), or activating barrier-protective cytokines through the AhR–IL-22 axis (strain L21) (Chen et al., 2025). In contrast, WIS43 exerted multi-level protective effects in DSS-induced colitis, simultaneously improving clinical parameters (DAI, body weight, colon length), reducing histopathological damage, and significantly suppressing key pro-inflammatory cytokines (TNF-*α*, IL-6, IL-1β) in both serum and colonic tissues. Importantly, WIS43 was directly compared with both a reference probiotic strain (Lpc-37) and the standard drug mesalazine—an evaluation that is largely absent from prior studies on *L. paracasei*. At the microbiota level, most prior investigations relied on short-read sequencing, which limits taxonomic resolution to the genus or high-level OTU level and prevents accurate identification of functional species. Using full-length 16S rRNA sequencing, our study resolved species-level changes and demonstrated that WIS43 selectively enriched several well-characterized beneficial Lactobacillus members—*L. johnsonii*, *L. taiwanensis*, *L. gasseri,* and *L. reuteri*—all known for their barrier-protective and immunomodulatory activities. Furthermore, WIS43 uniquely increased *G. urolithinfaciens*, a urolithin-producing species associated with mitochondrial protection and anti-inflammatory activity—an effect rarely reported for other *L. paracasei* strains. This coordinated enrichment of multiple functional species suggests that WIS43 acts not only as a probiotic itself but also as a microbial ecosystem modulator that promotes a consortium of protective commensals. Beyond microbiota modulation, WIS43 also exhibited superior gastrointestinal survival, stronger pathogen antagonism, and more potent antioxidant activity than the reference strain Lpc-37, further reinforcing its functional advantages as a probiotic (Supplementary Figure S1; Supplementary Table S2; Figure 2C). our findings highlight the therapeutic value of WIS43 in ameliorating UC and provide experimental evidence supporting its development as a next-generation probiotic intervention.

Overall, our findings highlight the therapeutic value of WIS43 in ameliorating UC and provide experimental evidence supporting its development as a next-generation probiotic intervention. Future studies should further dissect its precise mechanistic pathways—including its immunomodulatory targets and microbiota-mediated functions—and comprehensively evaluate its long-term safety, and stability under gastrointestinal conditions.

## 5 Conclusion

In summary, this study characterized a novel *L. paracasei* strain, WIS43, isolated from human breast milk, which exhibited superior *in vitro* properties—including antioxidant capacity and gastrointestinal tolerance—compared to the commercial strain Lpc-37. *In vivo* experiments demonstrated that WIS43 significantly alleviated DSS-induced UC in mice by preserving colon length, reducing DAI scores, and suppressing pro-inflammatory cytokines (TNF-α, IL-1β, and IL-6). Crucially, WIS43 intervention restructured the gut microbiota, characterized by the enrichment of potential beneficial taxa such as *L. gasseri* and the suppression of colitis-associated pathobionts. These findings provide strong evidence that *L. paracasei* WIS43 exerts its protective effects through the synergistic modulation of immune responses and gut microbiota composition, suggesting its potential as a promising functional food ingredient or probiotic therapeutic for the management of inflammatory bowel diseases.

## Data Availability

The datasets presented in this study can be found in online repositories. The data can be found here: https://www.ncbi.nlm.nih.gov/, PRJNA1403732.
