# Supplementary material for: Lactobacillus paracasei WIS43 alleviates DSS-induced colitis by modulating gut microbiota and suppressing inflammation
Source: Front Microbiol. 2026 Jan 29;16:1721585. doi: 10.3389/fmicb.2025.1721585 (PMC12900382; doi:10.3389/fmicb.2025.1721585)
Supplement: Supplementary file 1 [file Table_1.DOCX]

Supplementary Material

# Supplementary Method

**Utilization of Different Carbohydrates by *Lactobacillus paracase*i WIS43**

The Lactobacillus paracasei WIS43 strain was first activated by streaking onto MRS agar plates and incubating anaerobically at 37°C for 48 hours. Well-isolated single colonies were then selected and suspended in 2 ml of sterile saline and vortexed thoroughly to obtain a uniform bacterial suspension.For optical density measurement, an aliquot of the suspension was diluted with 5 ml of sterile saline, and the OD_600_ value was recorded. Meanwhile, a volume of the original bacterial suspension corresponding to twice that used for OD measurement was mixed with 10 ml of API medium (API 50 CH carbohydrate identification kit, bioMérieux) and mixed thoroughly.

To prepare the incubation system, sterile deionized water was added to the wells at the bottom of the incubation tray to maintain a humid environment. One API 50 CH test strip was placed in the tray. Approximately 115 µl of the bacterial and medium mixture was added to each cupule using a micropipette.The upper part of each tube was left unsealed to allow an anaerobic microenvironment to form within the tube. The incubation tray was placed in a 37°C incubator. Color changes in each reaction cupule were examined after 24 hours and again after 48 hours. Carbohydrate fermentation profiles were interpreted according to the manufacturer’s instructions, and the results are summarized in Supplementary Table S1.

**Antibacterial Activity Assay of *Lactobacillus paracasei* WIS43**

The antibacterial activity of *Lactobacillus paracasei* WIS43 was evaluated using the Oxford cup diffusion method. Seven common pathogenic indicator strains were selected for testing: *Escherichia coli* ATCC25922, *Staphylococcus aureus* ATCC25923, *Pseudomonas aeruginosa* ATCC27853, *Enterococcus faecalis* ATCC29212, *Shigella flexneri* CICC21534, *Cronobacter sakazakii* CICC21560, and *Salmonella typhimurium* CICC21513.

Each indicator strain was inoculated (1%, v/v) into BHI broth and incubated at 37°C for 16 h. The bacterial cultures were then diluted to an OD_600_ of approximately 0.025 and evenly spread onto pre-warmed BHI agar plates.

WIS43 and the reference strain Lpc-37 were cultured in MRS broth at 37°C for 24 h. The cultures were centrifuged, washed, and resuspended in sterile saline. Oxford cups were placed on the surface of the indicator lawn, and 200 µl of the bacterial suspension was added to each cup. Sterile MRS broth was used as the negative control.

The plates were incubated at 4°C for 2 h to allow diffusion, followed by incubation at 37°C for 24 h. Antibacterial activity was assessed by measuring the diameter of the inhibition zones around each Oxford cup.

**Simulated Gastrointestinal Fluid Tolerance Assays**

The tolerance of Lactobacillus paracasei WIS43 to gastrointestinal conditions was evaluated using simulated gastric fluid (SGF) and simulated intestinal fluid (SIF). SGF was prepared by dissolving NaCl (0.1 g) and pepsin (0.175 g) in 50 mL of distilled water, adjusting the pH to 2.5, and sterilizing the solution through a 0.22 μm membrane. SIF was prepared by dissolving trypsin (6.8 g) and bile salts (10 g) in distilled water, adjusting the pH to 6.8 with 0.1 mol/L NaOH, diluting to 1 L, and filtering through a 0.22 μm membrane.

Glycerol-preserved bacterial stocks were activated by inoculation into MRS broth (1% inoculum) and incubated anaerobically at 37°C for 16 h. The cultures were harvested by centrifugation (6000 rpm, 10 min), washed twice with sterile saline, and resuspended.

For gastric tolerance testing, 3 mL of SGF was mixed with the bacterial suspension to a final volume of 10 mL, and an aliquot was immediately sampled for viable counting on MRS agar (N₀). The mixture was then incubated at 37°C for 3 h, followed by viable counting (N₃ₕ). Intestinal tolerance was assessed similarly by replacing SGF with SIF, incubating the mixture at 37°C for 3 h, and determining viable counts before and after exposure. Survival rates were calculated as (N₃ₕ / N₀) × 100%. All assays were performed in triplicate, and L. paracasei Lpc-37 was included as a reference strain.

# Supplementary Table S1.Utilization Test Results of Carbohydrates by WIS43

| **Substrate** | ****Result**** | **Substrate** | ****Result**** |
| --- | --- | --- | --- |
| **Control** | - | **Esculin** | - |
| **Glycerol** | + | **Salicin** | + |
| **Erythritol** | - | **D-Cellobiose** | + |
| **D-Arabinose** | - | **D-Maltose** | + |
| **L-Arabinose** | - | **D-Lactose** | - |
| **D-Ribose** | + | **D-Melezitose** | - |
| **D-Xylose** | - | **D-Sucrose** | + |
| **L-Xylose** | - | **D-Trehalose** | + |
| **Adonitol / Ribitol** | - | **Inulin** | + |
| **Methyl $ \beta $-D-xylopyranoside** | - | **D-Raffinose** | + |
| **D-Galactose** | + | **D-Raffinose** | - |
| **D-Glucose** | + | **Starch** | - |
| **D-Fructose** | + | **Glycogen** | - |
| **D-Mannose** | - | **Xylitol** | - |
| **L-Sorbose** | - | **D-Gentiobiose** | + |
| **L-Rhamnose** | - | **D-Isomaltose** | + |
| **Dulcitol / Galactitol** | - | **D-Lyxose** | - |
| **Inositol** | - | **D-Tagatose** | + |
| **D-Mannitol** | + | **D-Fucose** | - |
| **D-Sorbitol** | + | **L-Fucose** | - |
| **Methyl -α-D-mannopyranoside** | - | **D-Arabitol** | - |
| **Methyl -α-D-glucopyranoside** | + | **L-Arabitol** | - |
| **N-Acetylglucosamine** | + | **Gluconate** | - |
| **Amygdalin** | + | **Potassium 2-ketogluconate** | - |
| **Arbutin** | + | **Potassium 5-ketogluconate** | - |

# Supplementary Table S2.Inhibitory ability of Lactobacillus paracasei supernatant against pathogens

| Pathogenic bacteria | WIS43 (mm) | Lpc-37 (mm) |
| --- | --- | --- |
| *Cronobacter sakazakii* | 21.70 ± 2.65 | 20.45 ± 2.56 |
| *Staphylococcus aureus* | 13.38 ± 1.84 | 13.12 ± 0.65 |
| *Pseudomonas aeruginosa* | 18.59 ± 1.08 | 18.78 ± 0.63 |
| *Shigella flexneri* | 19.25 ± 1.98 | 18.91 ± 1.54 |
| *Enterococcus faecalis* | 18.20 ± 0.68 | 16.50 ± 1.70 |
| *Escherichia coli* | 24.51 ± 1.57 | 22.94 ± 1.24 |
| *Salmonella typhimurium* | 23.48 ± 0.97 | 18.21 ± 1.35 |

# Supplementary Table S3. Antioxidant activities of Lactobacillus paracasei WIS43 and Lpc-37

| **Strain** | **DPPH Radical Scavenging Activity (%)** | | **•OH Radical**  **Scavenging Activity (%)** | | **Total Antioxidant Capacity (mM FeSO₄ Equivalent)** | |
| --- | --- | --- | --- | --- | --- | --- |
|  | Cell Lysate | Supernatant | Cell Lysate | Supernatant | Cell Lysate | Supernatant |
| ***L. paracasei* Lpc-37** | 18.94 | 80.74 | 40.39 | 53.99 | 0.0696 | 0.2176 |
| ***L. paracasei* WIS43** | 19.54 | 79.76 | 32.44 | 54.87 | 0.0725 | 0.6266 |

# Supplementary Figures S1


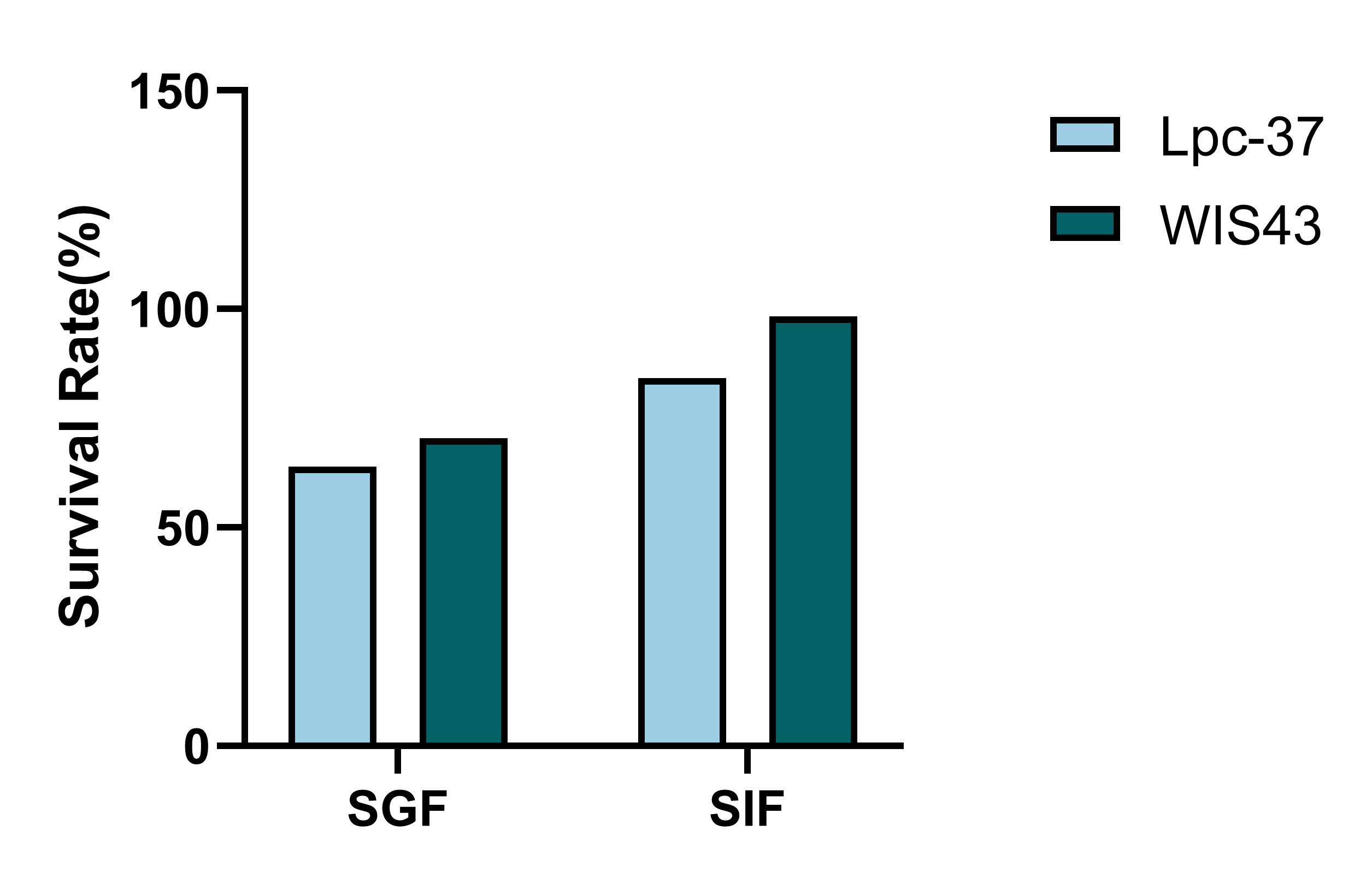


**Supplementary Figure S1.** Survival rate of Lactobacillus paracasei Lpc-37 and WIS43 strains in simulated gastric fluid (SGF) and simulated intestinal fluid (SIF) in vitro. The results show that the survival rates of both strains were higher in simulated intestinal fluid than in simulated gastric fluid, and the WIS43 strain exhibited a slightly higher survival rate than the Lpc-37 strain in both simulated digestive fluids.
